# Abnormal Paraplegin Expression in Swollen Neurites, τ- and α-Synuclein Pathology in a Case of Hereditary Spastic Paraplegia SPG7 with an Ala510Val Mutation

**DOI:** 10.3390/ijms161025050

**Published:** 2015-10-21

**Authors:** Dietmar R. Thal, Stephan Züchner, Stephan Gierer, Claudia Schulte, Ludger Schöls, Rebecca Schüle, Matthis Synofzik

**Affiliations:** 1 Laboratory of Neuropathology—Institute of Pathology, Center of Clinical Research, University of Ulm, Helmholtzstraße 8/1, D-89081 Ulm, Germany; 2 Department of Neuroscience, Katholieke Universiteit Leuven, B-3000 Leuven, Belgium; 3 Dr. John T. Macdonald Foundation Department of Human Genetics University of Miami Miller School of Medicine, Miami, FL 33136, USA; E-Mails: SZuchner@med.miami.edu (S.Z.); rebecca.schuele-freyer@uni-tuebingen.de (R.S.); 4 John P. Hussman Institute for Human Genomics, University of Miami Miller School of Medicine, Miami, FL 33136, USA; 5 Outpatient Praxis for Neurology, D-89407 Dillingen, Germany; E-Mail: dr.gierer@neurologie-dillingen.de; 6 Department of Neurodegenerative Diseases, Hertie-Institute for Clinical Brain Research, Hoppe-Seyler-Strasse 3, University of Tübingen, 72077 Tübingen, Germany; E-Mails: claudia.schulte@uni-tuebingen.de (C.S.); ludger.schoels@uni-tuebingen.de (L.S.); 7 German Research Center for Neurodegenerative Diseases (DZNE), University of Tübingen, 72076 Tuebingen, Germany

**Keywords:** SPG7, neurofibrillary tangles, tau, spastic paraplegia, ataxia, spastic ataxia, coiled bodies, Lewy bodies, paraplegin

## Abstract

Mutations in the *SPG7* gene are the most frequent cause of autosomal recessive hereditary spastic paraplegias and spastic ataxias. Ala510Val is the most common *SPG7* mutation, with a frequency of up to 1% in the general population. Here we report the clinical, genetic, and neuropathological findings in a homozygous Ala510Val SPG7 case with spastic ataxia. Neuron loss with associated gliosis was found in the inferior olivary nucleus, the dentate nucleus of the cerebellum, the substantia nigra and the basal nucleus of Meynert. Neurofilament and/or paraplegin accumulation was observed in swollen neurites in the cerebellar and cerebral cortex. This case also showed subcortical τ-pathology in an unique distribution pattern largely restricted to the brainstem. α-synuclein containing Lewy bodies (LBs) were observed in the brainstem and the cortex, compatible with a limbic pattern of Braak LB-Disease stage 4. Taken together, this case shows that the spectrum of pathologies in SPG7 can include neuron loss of the dentate nucleus and the inferior olivary nucleus as well as neuritic pathology. The progressive supranuclear palsy-like brainstem predominant pattern of τ pathology and α-synuclein containing Lewy bodies in our SPG7 cases may be either coincidental or related to SPG7 in addition to neuron loss and neuritic pathology.

## 1. Introduction

Mutations in the *SPG7* gene are the most frequent causes of autosomal recessive Hereditary Spastic Paraplegias (HSP) and spastic ataxias [1]. The most common mutation is the p.Ala510Val mutation, with a carrier frequency of up to 1% in the general population [2,3]. Paraplegin, the gene product of *SPG7*, is a mitochondrial metalloprotease located at the inner mitochondrial membrane [4]. Multiple mutations in *SPG7* have been described to cause HSPs and spastic ataxias, and an important role of this protein in neuronal mitochondrial function is assumed also clincially [2,3].

However, the neuropathology underlying the HSP with *SPG7* mutation (=SPG7) remains to be ascertained, as only the neuropathology of one single SPG7 case (with a p.Arg470Gln *SPG7* mutation) has been reported so far [3]. In this single case, white matter changes in the spinal cord in addition to the axonal swellings in the cerebellum (so called axonal torpedoes) were described but no τ or α-synuclein aggregates. To our knowledge, no report about the Ala510Val neuropathology exists.

Here, we demonstrate neuronal degeneration in the dentate nucleus and the inferior olivary nucleus and an unexpected brainstem τ pathology in a SPG7 case with a homozygous p.Ala510Val mutation.

## 2. Results and Discussion

### 2.1. Case Report

**Figure 1:**
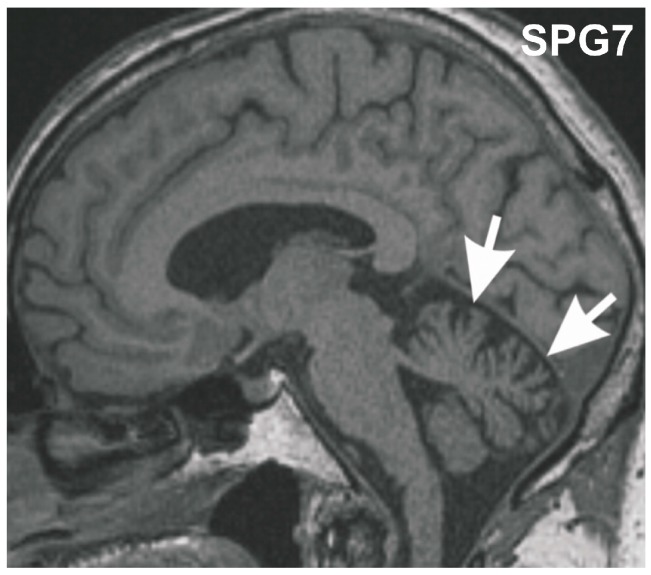
Midsagittal T1 weighted MRI of the SGP7 index patient at age 70 years. The MRI shows mild vermian cerebellar atrophy (arrows).

We report a case with a homozygous p.Ala510Val mutation presenting with the clinical features of a late-onset spastic spinocerebellar ataxia. The index family provided consent to this study, which was approved by the ethic committees in Ulm and Tübingen. The Caucasian man developed a slowly-progressive spastic-ataxic gait disorder starting at age 59 years accompanied by mild dysdiadochokinesis, mild dysmetria in finger-nose test as well as mild dysmetria in the heel-shin slide, corresponding to a mild cerebellar atrophy (for MRI, Figure 1), followed by chronic progressive external ophthalmoplegia (cPEO) in later years. This phenotypic combination is well established for SPG7 [1,5]. At age 68 years he started to use a cane, since age 69 years a walking frame. Upper limb deficits did not lead to major impairments in daily life. Clinical follow-up examinations up to his death at age 70 years (to coronary heart disease) showed signs of mild cognitive impairment (MCI) with a Mini-Mental State score of 23/30 points, but no related functional impairments in daily living, *i.e.*, no dementia. Signs of Parkinsonism were not observed, in particular no signs of progressive supranuclear palsy (PSP). His sister was similarly affected with progressive spastic-ataxic gait disorder and cPEO, starting at age 50 years. She also did not exhibit any signs of Parkinsonism, and was cognitively intact. No neurological disturbances were reported for the patient’s parents, indicating an autosomal recessive mode of inheritance.

### 2.2. Whole Genome Sequencing

**Figure 2:**
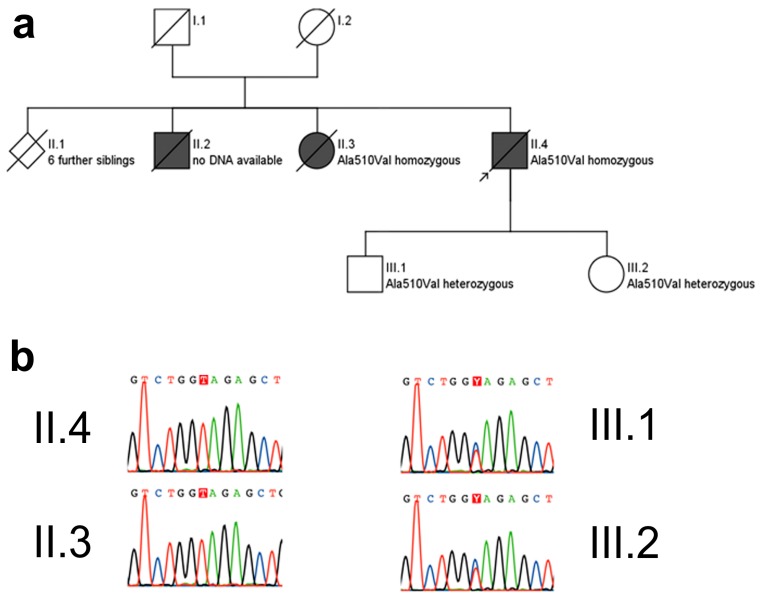
Pedigree (**a**) and electropherograms (**b**) of the p.Ala510Val SPG7 family. The p.Ala510Val *SPG7* variant was observed in a homozygous state in both affected subjects for whom DNA was available, namely in the index patient (arrow) and his sister. She was similarly affected with progressive spastic-ataxic gait disorder and cPEO, starting at age 50 years. The unaffected children of the index patient carried this mutation in a heterozygous state. Pedigree symbols for **a**: squares: male; circle: female; diamond: gender not specified; black filled symbols: affected by disease; white symbols: healthy; symbols cross by a line: deceased. Color codes for **b**: G (guanin): black, T (thymine): red, C (cytosine): blue, A (adenine): green.

Whole exome sequencing yielded six genes carrying variants qualifying the filter criteria (Table S1). Among these six genes, *SPG7* was the only one known to be associated with the phenotype. The identified homozygous c.1529 C>T, p.Ala510Val mutation in *SPG7* is a known mutational hotspot in *SPG7* [1,4]. This mutation was also confirmed in the affected sister, thus showing segregation with disease, and present in heterozygous state in his two unaffected children, thus indicating location of the variants in *trans* (Figure 2).

### 2.3. Neuropathology

**Figure 3:**
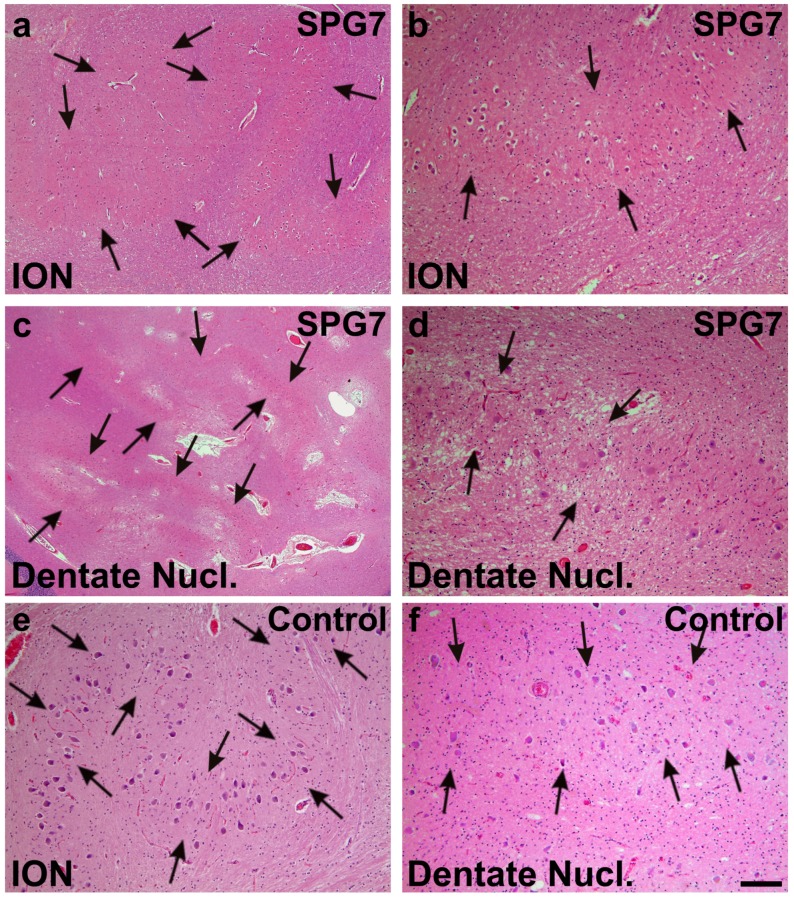
Moderate neuron loss and gliosis in the inferior olivary nucleus (ION) (arrows in **a**,**b**) and in the dentate nucleus (dentate nucl.) of the SPG7 case (arrows in **c**,**d**); **a**,**c** show overviews of the ION (**a**) and the dentate nucleus (**c**), whereas at increased magnification (**b**,**d**) a moderately reduced neuron frequency could be observed when comparing with control cases as depicted for control case number three (**e**,**f**). Arrows in **e**,**f** indicate normal neuron densities in ION (**e**) and the dentate nucleus (**f**). Calibration bar in **f** valid for **a**: 320 µm; **c**: 710 µm; **b**,**d**–**f**: 130 µm.

**Figure 4:**
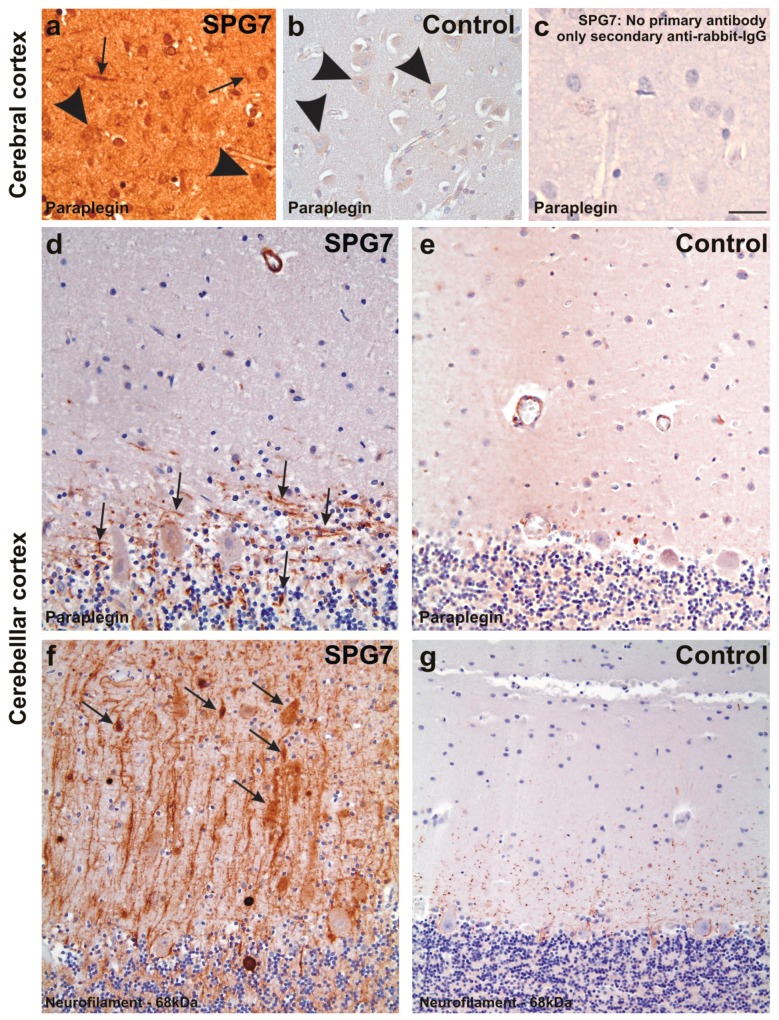
Paraplegin expression in the frontal neocortex of SPG7 case (**a**) and of a non-diseased control (case No. 2) (**b**); (**c**) depicts the negative control for the SPG7 case by omitting the primary antibody to document the specificity of the immunostainings. Despite an overall increased staining intensity in the SPG7 case that can be explained by varying staining intensities, neurons were slightly stained in both the SPG7 case and in the control (arrowheads in **a**,**b**), whereas neurites exhibited paraplegin only in the SPG7 case (arrows in **a**) and not in the healthy control brain. Similarly, neurites exhibit paraplegin in the Purkinje cell layer of the cerebellum in the SPG7 case (arrows in **d**) but not in the control (case number four) (**e**); Here only the perikarya of the Purkinje cells were labeled mildly (**e**); The presence of paraplegin positive neurites in the cerebellum was associated with high numbers of neurites accumulating 68 kDa neurofilaments. Some of these neurites appear swollen (arrows in **f**); In the control (case number four) there was no neuritic accumulation of the 68 kDa neurofilament protein (**g**); Calibration bar in **c** valid for **a**–**c**: 35 µm; **d**,**e**: 40 µm; **f**,**g**: 50 µm.

**Figure 5:**
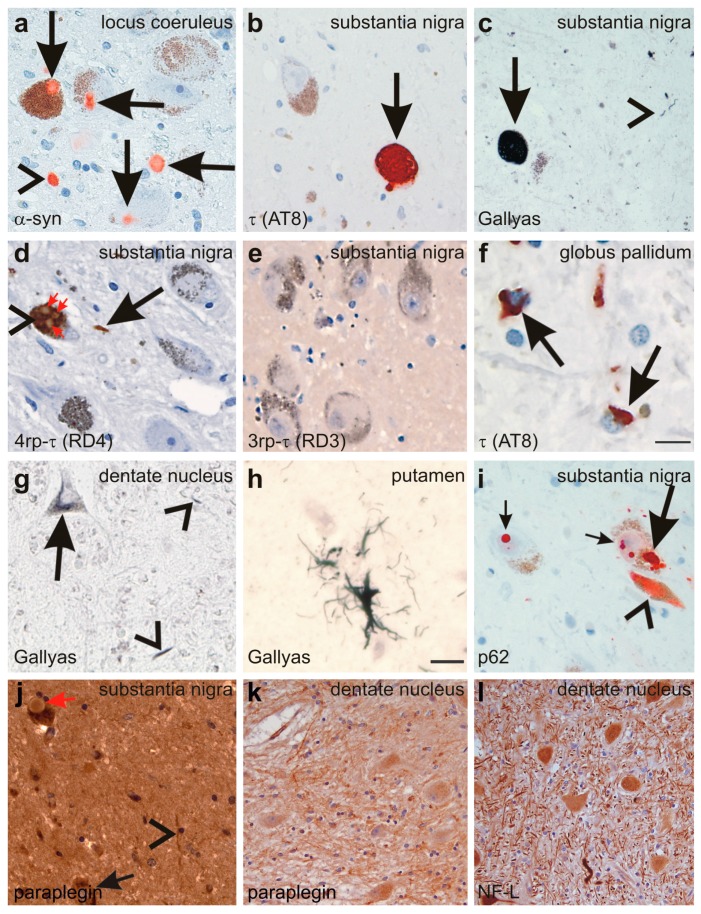
(**a**) Lewy-bodies (arrows) and Lewy neurites (arrowhead) are detected in the locus coeruleus with the anti-α-synunclein (α-syn) antibody; (**b**,**c**) NFTs are detected in the neurons of the substantia nigra with an antibody directed against abnormal phosphorylated τ-protein (arrow in **b**) as well as with the Gallyas silver method (arrow in **c**). Neuropil threads were also detected (arrowhead in **c**); (**d**,**e**) the abnormal τ-protein aggregates in the substantia nigra nerve cells (arrowhead in **d**) and in neuropil threads contained 4rp τ (arrows in **d**) but no 3rp τ (**e**); Lewy bodies (red arrows in **d**) occurring inside τ-positive NFTs (arrowhead in **d**) did not exhibit immunoreactivity with anti-τ antibodies such as anti-rp4 τ; (**f**) coiled bodies were detected with an antibody raised against abnormal phosphorylated τ-protein in the globus pallidum (arrows); (**g**) neuronal silver-stained inclusions (arrow) as well as neuropil threads (arrowheads) also occurred in the cerebellar dentate nucleus; (**h**) single glial inclusions with a tufted astrocyte-like pattern were detected in the putamen by Gallyas silver staining; (**i**) NFTs (arrowhead) and LBs (large and small arrows) were also detectable with an antibodies directed against p62 as documented in the substantia nigra; (**j**) Although paraplegin was detected in neurites (arrowheads) and neurons of the substantia nigra (arrow), Lewy bodies were not specifically labeled (red arrow); (**k**) neuritic accumulation of paraplegin was also observed in the dentate nucleus; and (**l**) antibodies against the 68 kDa subunit of neurofilaments also showed some neurites with neurofilament accumulation. Calibration bar in **f** valid for **a**–**f**, **i**–**l**: 30 µm; **g**,**h**: 15 µm.

Post-mortem brain autopsy demonstrated macroscopically a brain of 1410 g with a slightly depigmented substantia nigra and a cerebellum with a small cerebellar vermis in line with vermian atrophy seen in MRI-imaging (Figure 1). Microscopically, there was reduced neuronal cellularity with and accompanied fibrous gliosis in the substantia nigra, the basal nucleus of Meynert, the inferior olivary nucleus, and the cerebellar dentate nucleus (Figure 3, Table 1). Paraplegin was immunohistochemically detected mainly in neuronal cytoplasmata in the healthy control neocortex and cerebellar Purkinje cells (Figure 4a–e) as previously described [6], while it accumulated in neurites of the SPG7 case (in addition to a slight cytoplasmic staining), indicating changes in the intraneuronal distribution of paraplegin-exhibiting mitochondria in SPG7 (Figure 4a,b). These changes were most prominent in the cerebellum (Figure 4d,e, Table 1). Moreover, pronounced accumulation of neurofilaments in swollen neurites was observed in the SPG7 case, but not in the control cases (Figure 4f,g). In addition, LB pathology (Figure 5a) exhibiting a distribution pattern fulfilling the criteria for Braak-LBD (Lewy body disease: summarizes the pathological spectrum of Parkinson’s disease and dementia with Lewy bodies) stage 4 was found (Table 1). Moreover, a unique distribution pattern of fibrillar, Gallyas-, and τ-positive neurofibrillary tangles (NFTs), and neuropil threads was observed with dissemination into the substantia nigra (Figure 5b–e), basal ganglia, pons, midbrain, and medulla oblongata. Single NFTs and neuropil threads were also observed in the dentate nucleus of the cerebellum (Figure 5g) whereas the central (primary motor) cortex did exhibit neither τ nor α-synuclein pathology nor evident neuron loss (Table 1). Oligodendroglial τ-containing coiled bodies (Figure 5f) were seen in a subcortical distribution pattern (Table 1), glial τ inclusions with a pattern similar to tufted astrocytes occurred in the basal ganglia (Figure 5h). The τ-lesions contained four-repeat (4rp) τ while 3rp τ was not detected (Figure 5d,e), indicating that the SPG7 related subcortical τ-pathology represents a 4rp tauopathy. The brainstem distribution of the τ-lesions and LBs (Table 1) might explain a nuclear contribution to the clinically observed ophthalmoparesis. Paraplegin did not accumulate in these inclusions (Figure 5j). No TDP43 aggregates were found in this case. Coincidental primary age-related tauopathy (PART)-related pathology [7] was restricted to NFTs in the transentorhinal regions representing Braak-NFT tangle stage I. AD-related NFT-pathology in control cases numbers three and five did not exhibit similar lesions as in the SPG7 case. Amyloid plaques or cerebral amyloid angiopathy were not observed in the SPG7 case, demonstrating the absence of Alzheimer’s disease (AD)-related pathology [8].

There was no significant white matter damage in the brain of our SPG7 case.

**Table 1:** Distribution of neuron loss and astrogliosis, neuritic pathology as detected by anti-paraplegin or anti-NF-L immunohistochemistry, NFT, τ-, and α-synuclein-pathology.

Region	Obvious Neuron Loss/Astrogliosis (H&E)	Swollen Neurites (NFP 68, Paraplegin)	Gallyas	τ-Pathology	α-Synuclein Pathology
*Cortex*					
Frontal cortex (A 6)	−	+	−	−	−
Parietal cortex (A 7)	−	n.a.	−	−	−
Central cortex (A 1–5)	−	n.a.	−	−	−
Temporal cortex (A 36)	−	n.a.	−	−	−
Occipital cortex (A 17–19)	−	n.a.	−	−	LN
Cingulate gyrus (A 24)	−	n.a.	−	−	LB
Entorhinal region (A 28)	−	n.a.	−	−	−
Transentorhinal region (A 35)	−	n.a.	NFT, NT	NFT, NT	−
Insular Cortex	−	n.a.	−	−	−
*Hippocampus*					
CA1-subiculum	−	n.a.	−	−	−
CA2	−	n.a.	−	−	−
CA3	−	n.a.	−	−	−
CA4	−	n.a.	−	−	−
Dentate Gyrus	−	n.a.	−	−	−
*Basal ganglia*					
Globus pallidus ^+^	−	n.a.	NFT, NT, CB	NFT, NT, CB	LB, LN
Putamen ^+^	−	n.a.	NFT, NT, CB, AG	NFT, NT, CB, AG	LB, LN, IC-Granules
Caudate nucleus ^+^	−	n.a.	NFT, NT	NFT, NT	LB, LN, IC-Granules
Basal nucleus of Meynert	++	n.a.	NFT, NT	NFT, NT	LB, LN, IC-Granules
Amygdala	−	n.a.	NFT, NT	NFT, NT	LB, LN
*Hypothalamus*					
Mammillary body	−	n.a.	−	−	−
Supramammillary nucleus	−	n.a.	NFT, NT	NFT, NT	LB, LB, IC-Granules
Tuberomammillary nucleus	−	n.a.	−	−	LB, LB, IC-Granules
Lateral mammillary nucleus	−	n.a.	−	−	LB, LB, IC-Granules
Lateral hypothalamic area	−	n.a.	NFT, NT	NFT, NT	LB, LB, IC-Granules
*Thalamus*					
Pulvinar	−	n.a.	NFT, NT	NFT, NT	LB, LB
Lateral genucilate body	−	n.a.	−	−	−
Medial geniculate body	−	n.a.	−	−	−
*Midbrain*					
Substantia nigra ^+^	++	+	NFT, NT	NFT, NT	LB, LN
Nucleus ruber	−	−	−	−	−
Central gray	−	(+)	NFT, NT	NFT, NT	LB
Oral raphe nucleus	−	−	NFT, NT	NFT, NT	LB
Colliculi inferior	−	+	NT	NT	LB
Deep mesencephalic nucleus	−	n.a.	NFT, NT	NFT, NT	LB
rostral interstitial nucleus of the medial longitudinal fascicle *	−	n.a.	NT	NT	−
Interstitial nucleus of Cajal *	−	n.a.	NFT, NT	NFT, NT	−
*Pons*					
Locus coeruleus	(+)	−	NFT, NT	NFT, NT	LB
Oral raphe nucleus	−	(+)	NFT, NT	NFT, NT	−
Paramedial pontine reticular formation	−	(+)	NFT, NT	NFT, NT	−
Pontine nuclei	−	+	NFT, NT	NFT, NT	−
Parabrachial nuclei	−	−	NT	NT	−
*Medulla oblongata*					
Vagal nerve nucleus	−	n.a.	−	-	LB, LN
Inferior olivary nucleus ^+^	+	n.a.	NFT, NT	NFT, NT	−
Intermediate reticular zone	−	n.a.	NFT, NT	NFT, NT	LB, LN
Vestibular nerve nuclei	−	n.a.	NFT, NT	NFT, NT	−
*Cerebellum*					
Cerebellar cortex ^+^	(+)	++	−	−	−
Dentate nucleus ^+^	++	(+)	NFT, NT, CB	NFT, NT, CB	−

* = nuclei involved in eye movement; ^+^ = nuclei involved in motor function-coordination; n.a. = not assessed; − = no pathology; (+) = very mild pathology; + = mild pathology; ++ = moderate pathology; +++ = severe pathology; NFT = neurofibrillary tangle in neurons; NT = neuropil thread; CB = coiled bodies in oligodendrocytes; LB = Lewy body in neurons; LN = Lewy neurites; IC-granules = intracytoplasmic granules in neurons; AG = τ-positive astroglial cells.

### 2.4. Discussion

Our findings shed new light on the neuropathological degeneration associated with SPG7 disease and the extent of brain regions affected by this increasingly recognized condition [1,2,5,8]. Specifically, we show (1) neuron loss and associated gliosis in the inferior olivary nucleus, the cerebellar dentate nucleus, the substantia nigra and the basal nucleus of Meynert explaining spastic ataxia; (2) neuritic changes in the cerebellum, brainstem and cortex with accumulation of paraplegin in these neurites; and (3) a progressive supranuclear palsy (PSP)-like pattern of 4rp τ, which is distinct from other known tauopathies as it is mainly restricted to the brainstem and the basal ganglia. Brainstem τ pathology is known in the brainstem type of PSP with Parkinsonism, *i.e.*, the Richardson’s syndrome [9]. However, non-AD tauopathies, including PSP, CBD, and Guam-disease [10,11,12] usually show significant cortical pathology even the brainstem variant of PSP [9], which was not seen in our SPG7 case. In PSP, tufted astrocytes also occur in the brainstem [13]. Accordingly, it is tempting to speculate that the tauopathy in our cases is specifically related to SPG7. The occurrence of the τ pathology in brainstem nuclei serving saccadic functions in eye movement regulation (e.g., rostral interstitial nucleus of the medial longitudinal fascicle and the interstitial nucleus of Cajal [14]) might explain parts of the clinical phenotype, especially a possible additional nuclear contribution to the clinical symptom of cPEO. Since these nuclei were not affected by LBs it is tempting to speculate that cPEO in our SPG7 case results, at least in part, from τ pathology. A further (indirect) argument for the involvement of τ in SPG7 is that we see accumulation of paraplegin in neurites. Since paraplegin is a mitochondrial protein [4] accumulation of paraplegin in neurites might represent an accumulation of mitochondria in these neurites, which may result from a failure axonal transport regulated by the τ protein [15,16]. Alternatively, a co-existing brainstem restricted variant of PSP without any neocortical τ lesions cannot be ultimately excluded.

Given (i) the function of *SPG7*/paraplegin as a mitochondrial metalloprotease located at the inner mitochondrial membrane, with mutations leading to mitochondrial network and DNA damage [5,17], and given the fact (ii) that mitochondrial damage is capable of inducing τ-pathology (e.g., Guam-/PSP-like τ-pathology) [18,19]; and given (iii) that our case with a mutation in *SPG7*/paraplegin exhibits significant τ-pathology in an unusual distribution pattern, it is worth speculating that this subcortical tauopathy is possibly triggered by mitochondrial dysfunction caused by *SPG7* mutations. This possible functional relation between *SPG7*/paraplegin mutations and 4rp-τ pathology warrants further confirmation by larger genetico-neuropathological studies. Whether the observed LB pathology is part of this SPG7 disease pattern, or just restricted to coincidental LBD (as the distribution of the LBs fits to that of Braak-LBD stage 4) remains to be ascertained.

In addition to τ and α-synuclein pathology, neuron loss and neuritic pathology was observed in the cerebellum and also in distinct brainstem nuclei (Table 1). Neuritic pathology in the cerebellar cortex showed neuritic swellings with accumulation of the 68 kDa neurofilament subunit and/or paraplegin. None of them were observed in non-diseased controls. This pathology is in line with the description of axonal torpedos in the cerebellum in another a previously reported case with SPG7-associated neuropathology carrying a p.Arg470Gln mutation [3] and explains in concert with neuron loss in the dentate nucleus and the inferior olivary nucleus the clinical phenotype of ataxia. In our case we were limited to formaldehyde fixed brain tissue precluding biochemical analysis of paraplegin and neurofilament RNA-expression levels. As such, we cannot decide whether accumulation of paraplegin and neurofilaments is the results of overexpression or mislocation. Further studies using frozen tissue and a higher number of autopsy cases will be required to address this point as well as to clarify whether pathological paraplegin expression is specific for SPG7 or whether there is a SPG7-specific distribution pattern of paraplegin expression in neurites. Unfortunately, no spinal cord was available from our case to investigate whether white matter changes were present also in the spinal cord, as reported in the previous SPG7 case [3]. However, significant white matter degeneration was not observed in the brain of the SPG7 case. Oligodendroglial coiled bodies in the dentate nucleus and in the cerebellar white matter may indicate that altered oligodendrocytes may contribute to dentate nucleus degeneration and to neuritic pathology in the cerebellum as a part of an underlying tauopathy. Because our case did not exhibit signs of Parkinsonism there were not obvious clinical signs induced by the LB pathology.

In contrast to the findings in our SPG7 case, no τ and/or α-synuclein pathology was reported for the previously reported p.Arg470Gln mutation case [3]. This difference cannot be explained by subject age, as both cases were 70 years of age. Instead, the τ- and α-synuclein pattern observed in our p.Ala510Val case might be explained by genetic differences, namely either by the different *SPG7* mutation and/or by additional heterozygous risk alleles in other recessive ataxia or spasticity genes that are not pathogenic *per se*, but may act as genetic modifiers of *SPG7* dysfunction and pathology, e.g., the heterozygous *ATP13A2*, *CEP290*, *PNPLA6*, and *POLG* (mitochondrial DNA polymerase γ) variants observed in our patient (see Table S2). For example, similar LB pathology as in our case has been reported in cases with recessive *POLG* mutations [18]. However, it cannot be ultimately excluded that the τ and α-synuclein pathology in our SPG7 case represents simply coincidental pathology.

## 3. Experimental Section

### 3.1. Neuropathology

The brain was obtained at autopsy and fixed in phosphate buffered formalin for three weeks (post-mortem interval prior to autopsy: 96 h). After dissection of the circle of Willis, the brain stem and the cerebellum were dissected at the midbrain level. Then, ~1 cm thick frontal slabs of the hemispheres were cut. The brainstem and the cerebellum were cut into ~0.5 cm slabs perpendicular to the Meynert brainstem axis. Blocks from frontal, parietal, occipital, temporal, and pre- and postcentral cortices were embedded in paraffin, as well as blocks including the hippocampal formation, the hypothalamus, thalamus, basal ganglia, basal nucleus of Meynert and amygdala, midbrain with substantia nigra, pons, medulla oblongata, and cerebellum. Hematoxylin and eosin staining, as well as the Gallyas silver staining were used for neuropathological diagnosis. In addition immunohistochemistry with antibodies directed against abnormal τ-protein (AT-8, Thermo-Scientific—Pierce Biotechnology, Rockford, IL, USA, 1/1000) three-repeat τ (3rp-τ; RD3, 8E6/C11, Millipore, Temecula, CA, USA, 1/500, formic acid and microwave pretreatment), four-repeat τ (4rp-τ; RD4, 1E1/A6, Millipore, Temecula, CA, USA, 1/1000, formic acid and microwave pretreatment), α-synuclein (KM51, Leica Biosystems—Novocastra, Newcastle, UK, 1/40, formic acid pretreatment), amyloid β-protein (Aβ) (4G8, Covance, Dedham, MA, USA, 1/5000, formic acid pretreatment), p62 (clone number 3/p62 LCK ligand, BD Transduction Laboratories, Mountain View, CA, USA, 1:500), TDP43 (clone 2E2-D3, Novus Biologicals, Littleton, CO, USA, 1:2000, formic acid and microwave pretreatment), paraplegin (polyclonal rabbit, Acris Antibodies, San Diego, CA, USA, 1/100), and the 68 kDa subunit of neurofilaments (NF-L, SPM 204, Zytomed, 1/100, microwave pretreatment; 24 h at 22 °C) was performed. The primary antibodies were detected with biotinylated secondary antibodies and either the ABC-complex and visualized with 3.3-diaminobencidine or AEC or with the Dako REAL Alkaline phosphatase/RED detection system (DAKO, Glostrup, Denmark). Sections were mounted in Eukitt (O. Kindler; Freiburg, Germany). Positive and negative controls were performed in parallel. The sections were mounted in Eukitt (O. Kindler; Freiburg, Germany). To clarify pathological findings in the SPG7 case, five non-diseased control cases were studied for comparison (Table 2).

**Table 2:** List of cases. PMI = post-mortem interval until autopsy, m = male, f = female, Aβ = amyloid β-protein, AD = Alzheimer’s disease, AGD = argyrophilic grain disease, CERAD-Score = Consortium to establish a registry for Alzheimer’s disease score for neuritic plaque density, LBD = Lewy body disease, MTL = medial temporal lobe, NIA-AA = National Institute of aging and Alzheimer Association, NFT = neurofibrillary tangle in neurons.

Case Number	Age	Gender	Diagnosis	Aβ-Phase (MTL)	Braak-NFT Stage	CERAD-Score	NIA-AA Degree of AD	Braak-LBD Stage	Presence of Infarcts/Hemorrhages	PMI
1	70	m	SPG7	0	1	0	0	5	0	96
2	62	m	control	0	0	0	0	0	0	48
3	67	m	control	1	1	0	1	0	0	24
4	69	f	control	0	0	0	0	0	0	24
5	80	f	control (mild AD + AGD)	3	2	0	1	3	1	12
6	56	m	control	0	0	0	0	0	0	48

Phases of AD-related Aβ plaque pathology in the medial temporal lobe (Aβ-phase (MTL)), Braak-stages for neurofibrillary tangle (NFT) pathology (Braak-NFT stage) and for Lewy body (LB) pathology (Braak-LBD stage), the CERAD (Consortium to Establish a Registry for AD) score for neuritic plaques, and the degree of AD pathology (NIA-AA degree of AD) were determined as recommended [8,20,21,22,23].

Spinal cord, peripheral nerve, and skeletal muscle were not available for analysis.

### 3.2. Whole Genome Sequencing

The SureSelect Human All Exon 50 Mb kit (Agilent, Santa Clara, CA, USA) was used for in-solution enrichment and exome sequencing was performed using the Hiseq2500 instrument (Illumina, San Diego, CA, USA). Paired-end reads of 100 bp length were produced. BWA (v0.5.9rc1) and GATK (v1.4–37) software packages were used to align sequence reads to the reference and call variant positions. Data was then imported into the GEnomes Management Application (GEM.app) for annotation and analysis (PMID: 23463597). We filtered for homozygous or compound heterozygous variants using the following filter settings: coding, non-synonymous variants with minor allele frequency in NHLBI EVS6500 <2%, and number of families in GEM.app with the same variant <50 families, GERP score >2 and PhastCons score >0.5 and phyloP >0.75, read-depth >10, and at least moderate genotype quality (GQ > 50).

## 4. Conclusions

Our findings extend the pattern of pathological lesions involved in SPG7 disease: In addition to neuron loss in the inferior olivary nucleus and the dentate nucleus we found neuritic accumulation of neurofilaments and mitochondria, compatible with the preliminary idea of a possible failure of neuritic transport functions in SPG7. Additional τ and α-synuclein lesions, seen in our case may either represent SPG7-related lesions due to mitochondrial dysfunction or simply coincidental pathology in a quite unique pattern.

## Supplementary Materials

Supplementary materials can be found at http://www.mdpi.com/1422-0067/16/10/25050/s1.
