# Supplementary material for: Abnormal Paraplegin Expression in Swollen Neurites, τ- and α-Synuclein Pathology in a Case of Hereditary Spastic Paraplegia SPG7 with an Ala510Val Mutation
Source: Int J Mol Sci. 2015 Oct 21;16(10):25050–66. doi: 10.3390/ijms161025050 (PMC4632789; doi:10.3390/ijms161025050)
Supplement: Supplementary file 1 [file ijms-16-25050-s001.zip › ijms-101073-supplementary.pdf]

Supplementary Information

Supplementary comment: Interpretation of the POLG p.Asn468Asp variant. A heterozygous p.Asn468Asp *POLG* mutation was previously identified in this family, at that time misinterpreted to cause POLG disease as a single heterozygous variant, *i.e.*, as a “dominant” variant [1]. However, clinical follow-up examination of the family six years later led us to re-evaluation of the affection status in the then 52 years old daughter of the index case described here. Based on subtle findings of fatigue, generalized muscle pain, slight coordinative difficulties in demanding tasks, and slight peripheral neuropathy on nerve conduction studies we had classified her as affected at age 46. None of her symptoms, however, progressed, and no cPEO evolved. Cerebral MRI ruled out any subclinical brain atrophy or POLG-characteristic cerebral changes (such as hyperintense lesions in the thalamus or in the cerebellar white matter) [2]. Rather, her features could be well explained by the alternative diagnosis of a generalized chronic pain syndrome with depression. We therefore re-classified the mode of inheritance in this family as autosomal-recessive and initiated whole exome sequencing in the index patient to search for disease causing mutations beyond the known heterozygous *POLG* variant.

In line with this reclassification of the *POLG* variant as a recessive variant, also previous reports classified this variant only as a recessive mutation, *i.e.*, as disease causing only in a biallelic state in combination with a second pathogenic *POLG* mutation [3–5]. Some studies found this variant also as single heterozygous variant, *i.e.*, with no second mutation detected in *POLG*, but did not claim that this would suffice to cause disease [3,6,7]. Thus, in sum, in contrast to our report in 2009 [1] and after following-up our index family and reviewing the subsequent literature, we do not consider the p.Asn468Asp *POLG* mutation as a potential dominant mutation.

To rule out a second *POLG* mutation in our index case, undetected by whole exome sequencing, the complete *POLG* gene was sequenced by Sanger sequencing and gene dose was analyzed for copy number variants (CNVs). However, no second mutation was identified. Thus, there was no evidence that the disease of the patient was caused by recessive *POLG* mutations. In addition, also the clinical phenotype does not match with the phenotypes that have ever been described for POLG: no case of POLG has ever been described with spastic tetraparesis. In fact, even pyramidal tract damage in general is an exceptional feature in POLG disease [2].

However, we can of course not exclude that this single *POLG* mutation might have a modifying influence on the phenotype caused by *SPG7* p.Ala510Val. This might be the case, in particular, as *POLG* mutations also lead to increased mtDNA damage and accumulation of mtDNA deletions (like *SPG7* mutations), thus adding to the mitochondrial disease burden.

In contrast to the autopsy findings in our SPG7 case, no τ and/or α-synuclein pathology was reported for the p.Arg470Gln mutation case [8]. This difference cannot be explained by subject age, as both cases were 70 years of age. Instead, the τ- and α-synuclein pattern observed in our p.Ala510Val case might be explained by genetic differences, namely either by the different *SPG7* mutation and/or by additional heterozygous risk alleles in other recessive ataxia or spasticity genes that are not pathogenic per se, but may act as genetic modifiers of *SPG7* dysfunction and pathology, e.g., the heterozygous *ATP13A2*, *CEP290*, *PNPLA6*, and *POLG* (mitochondrial DNA polymerase γ) variants observed in our patient (see Table S2). For example, similar LB pathology as in our case has been reported in cases with recessive *POLG* mutations [9]. However, it cannot be excluded that the τ and α-synuclein pathology in our SPG7 case represents simply coincidental pathology.

Supplementary Table Legends:

Supplementary Table 1: Rare heterozygous genetic variants identified in the index SPG7 patient by whole exome sequencing. The SureSelect Human All Exon 50Mb kit (Agilent, Santa Clara, CA, USA) was used for in-solution enrichment and exome sequencing was performed using the Hiseq2500 instrument (Illumina, San Diego, CA, USA). Paired-end reads of 100 bp length were produced. BWA (v0.5.9rc1) and GATK (v1.4-37) software packages were used to align sequence reads to the reference and call variant positions. Data was then imported into the GEnomes Management Application (GEM.app) for annotation and analysis (PMID: 23463597). We filtered first for homozygous or compound heterozygous variants (detailed filter settings and variants). Among six genes carrying qualifying variants, *SPG7* was the only one known to be associated with the phenotype. The Ala510Val variant is a known mutational hotspot in *SPG7* [8,10].

Filter criteria: functional effect, coding, non-synonymous; frequency, minor allele frequency in NHLBI EVS6500 <2%, number of families in GEM.app with the same variant <50 families with the same variant; conservation, GERP score > 2 AND PhastCons score > 0.5 AND phyloP > 0.75; genotype quality, GQ > 50; read depth, read-depth > 10; known HSP/ATX genes are highlighted in red. GEM.app = GEnomes Management Application (GEM.app); EVS = Exome Variant Server 6500 exomes all from the NHLBI GO Exome Sequencing Project; PhastCons: conservation score which ranges from 0-1, with higher scores indicating a higher probably of conservation of the nucleotide; Gerp: Genomic Evolutionary Rate Profiling (GERP) score which ranges from −12.3 to 6.17, with 6.17 being the most conserved. Abbreviations of the MutationTaster predictions: D = disease-causing; N = polymorphism. Abbreviations from the Mutation assessor predictions: H = high, M = medium; L = low; N = neutral, SIFT, Sorting Tolerant From Intolerant; dbSNP ID = identification digit in the Single Nucleotide Polymorphism database (dbSNP)

Supplementary Table 2: To identify potential genetic modifiers of the neuropathological phenotype observed in the SPG7 index case we also filtered for heterozygous occurrence of variants (detailed filter settings and variants). Among the 164 heterozygous variants thus identified four affected known HSP or ataxia genes, including rare variants in *ATP13A2*, *CEP290*, *PNPLA6*, and *POLG*.

Filter criteria: functional effect, coding, non-synonymous; frequency, minor allele frequency in NHLBI EVS6500 <0.5%, number of families in GEM.app with the same variant <15 families with the same variant; conservation, GERP score > 2 OR PhastCons score > 0.5 OR phyloP > 0.75; genotype quality, GQ > 50; read depth, read-depth > 10; known HSP/ATX genes are highlighted in red.

GEM.app = GEnomes Management Application (GEM.app); EVS = Exome Variant Server 6500 exomes all from the NHLBI GO Exome Sequencing Project; PhastCons: conservation score which ranges from 0–1, with higher scores indicating a higher probably of conservation of the nucleotide; Gerp: Genomic Evolutionary Rate Profiling (GERP) score which ranges from −12.3 to 6.17, with 6.17 being the most conserved. Abbreviations of the MutationTaster predictions: D = disease-causing; N = polymorphism. Abbreviations from the Mutation assessor predictions: H = high, M = medium; L = low; N = neutral, SIFT, Sorting Tolerant From Intolerant; dbSNP ID = identification digit in the Single Nucleotide Polymorphism database (dbSNP)

References

1. Schulte, C.; Synofzik, M.; Gasser, T.; Schols, L. Ataxia with ophthalmoplegia or sensory neuropathy is frequently caused by POLG mutations. *Neurology* **2009**, *73*, 898–900.
2. Synofzik, M.; Srulijes, K.; Godau, J.; Berg, D.; Schols, L. Characterizing POLG ataxia: Clinics, electrophysiology and imaging. *Cerebellum* **2012**, *11*, 1002–1011.
3. Luoma, P.; Melberg, A.; Rinne, J.O.; Kaukonen, J.A.; Nupponen, N.N.; Chalmers, R.M.; Oldfors, A.; Rautakorpi, I.; Peltonen, L.; Majamaa, K.; *et al*. Parkinsonism, premature menopause, and mitochondrial DNA polymerase γ mutations: Clinical and molecular genetic study. *Lancet* **2004**, *364*, 875–882.
4. Woodbridge, P.; Liang, C.; Davis, R.L.; Vandebona, H.; Sue, C.M. POLG mutations in australian patients with mitochondrial disease. *Int. Med. J.* **2013**, *43*, 150–156.
5. Ylonen, S.; Ylikotila, P.; Siitonen, A.; Finnila, S.; Autere, J.; Majamaa, K. Variations of mitochondrial DNA polymerase γ in patients with Parkinson's disease. *J. Neurol.* **2013**, *260*, 3144–3149.
6. Blok, M.J.; van den Bosch, B.J.; Jongen, E.; Hendrickx, A.; de Die-Smulders, C.E.; Hoogendijk, J.E.; Brusse, E.; de Visser, M.; Poll-The, B.T.; Bierau, J.; *et al*. The unfolding clinical spectrum of POLG mutations. *J. Med. Genet.* **2009**, *46*, 776–785.
7. Tang, S.; Wang, J.; Lee, N.C.; Milone, M.; Halberg, M.C.; Schmitt, E.S.; Craigen, W.J.; Zhang, W.; Wong, L.J. Mitochondrial DNA polymerase γ mutations: An ever expanding molecular and clinical spectrum. *J. Med. Genet.* **2011**, *48*, 669–681.
8. Van Gassen, K.L.; van der Heijden, C.D.; de Bot, S.T.; den Dunnen, W.F.; van den Berg, L.H.; Verschuuren-Bemelmans, C.C.; Kremer, H.P.; Veldink, J.H.; Kamsteeg, E.J.; Scheffer, H.; *et al.* Genotype-phenotype correlations in spastic paraplegia type 7: A study in a large Dutch cohort. *Brain: J. Neurol.* **2012**, *135*, 2994–3004.
9. Betts-Henderson, J.; Jaros, E.; Krishnan, K.J.; Perry, R.H.; Reeve, A.K.; Schaefer, A.M.; Taylor, R.W.; Turnbull, D.M. α-Synuclein pathology and Parkinsonism associated with *POLG1* mutations and multiple mitochondrial DNA deletions. *Neuropathol. Appl. Neurobiol.* **2009**, *35*, 120–124.
10. Klebe, S.; Depienne, C.; Gerber, S.; Challe, G.; Anheim, M.; Charles, P.; Fedirko, E.; Lejeune, E.; Cottineau, J.; Brusco, A.; *et al*. Spastic paraplegia gene 7 in patients with spasticity and/or optic neuropathy. *Brain: J. Neurol.* **2012**, *135*, 2980–2993.
